# Congenital absence of left circumflex artery with right coronary dominance

**DOI:** 10.1002/ccr3.3457

**Published:** 2020-11-24

**Authors:** Bishika Pun, Amit Shrestha, Bipin Karki, Abhushan S. Tuladhar

**Affiliations:** ^1^ Department of Radiology Om Hospital and Research Center Kathmandu Nepal; ^2^ Department of Critical Care Medicine Om Hospital and Research Center Kathmandu Nepal; ^3^ Department of Radiology Nepal Medical College & Teaching Hospital Kathmandu Nepal

**Keywords:** chest pain, congenital coronary artery anomalies, left circumflex artery

## Abstract

Absent left circumflex coronary (LCX) artery though rare and benign should be considered in patients with chest pain and differentiated from atherosclerotic coronary artery disease for better management & prognosis.

## Graphical abstract

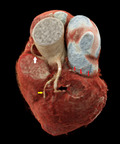

## 1 CASE SUMMARY

An 80‐year‐old female with hypertension presented in ER with history of chest pain on exertion, giddiness, and palpitation. On examination her pulse was 62 beats per minute, Respiratory rate 20 breaths per minute, blood pressure was 110/70 mm of Hg & oxygen saturation of 97%. Cardiovascular examination and initial electrocardiogram showed no abnormalities or ischemic changes. Routine blood work, cardiac enzymes, and chest X‐ray were within normal limits. Echocardiogram showed mitral and tricuspid regurgitation with left ventricle ejection fraction of 55%. Stress (treadmill) test was not done in this patient.

**FIGURE 1:**
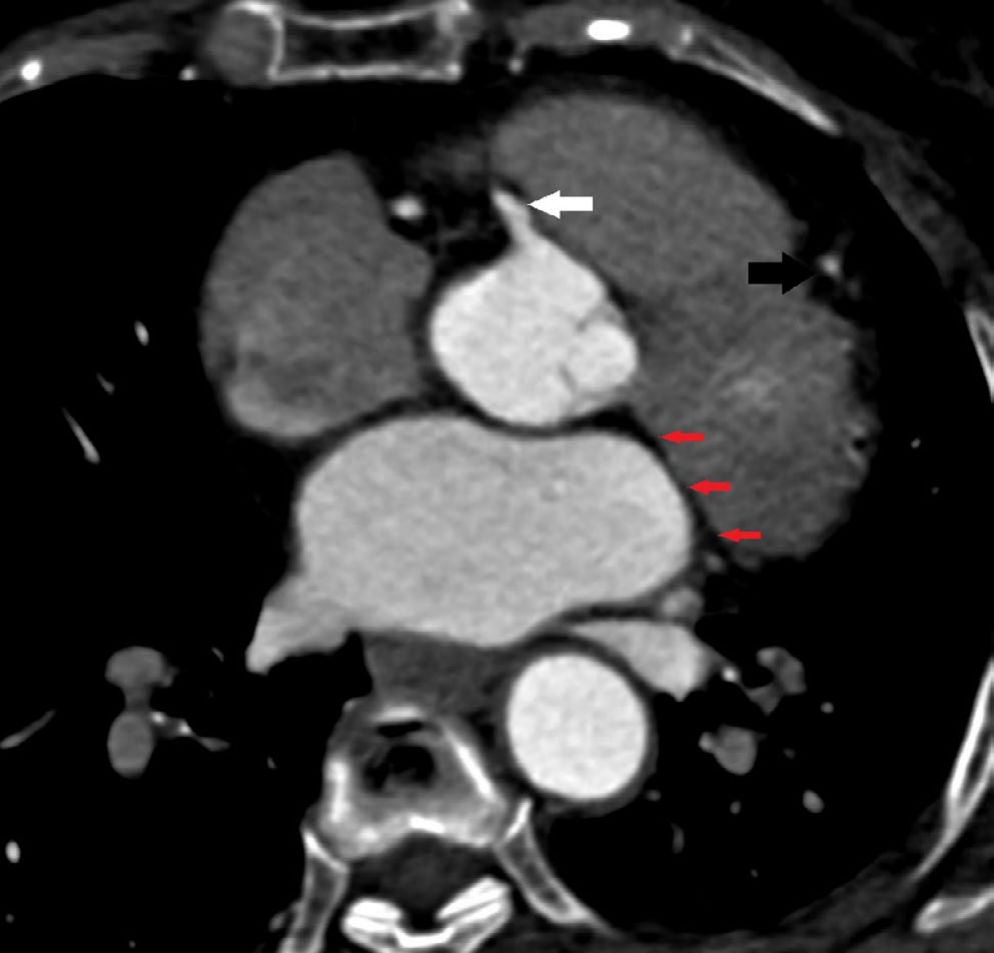
Axial image shows absent LCX in atrioventricular groove (red arrows), slightly prominent RCA (white arrow), LAD (black arrow) in interventricular groove

**FIGURE 2:**
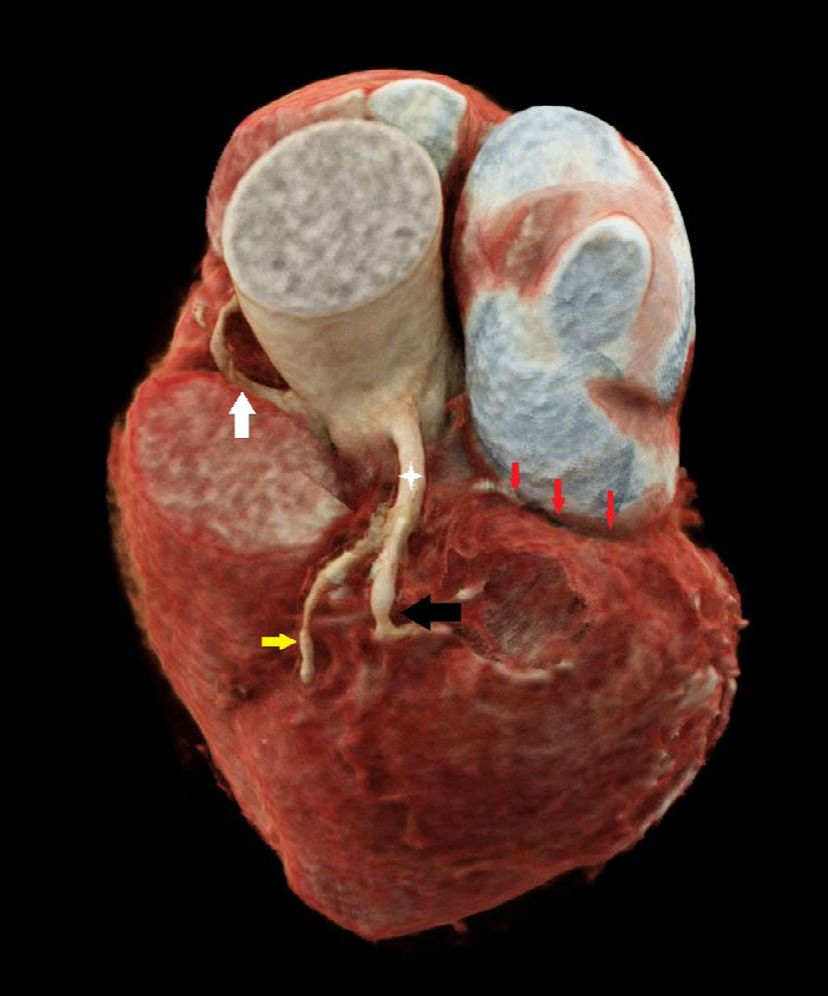
Cinematic volume‐rendered image shows LAD (black arrow) arising from LCA (white star). Diagonal branch (yellow arrow) arising from LAD, absent LCX (red arrows)

**FIGURE 3:**
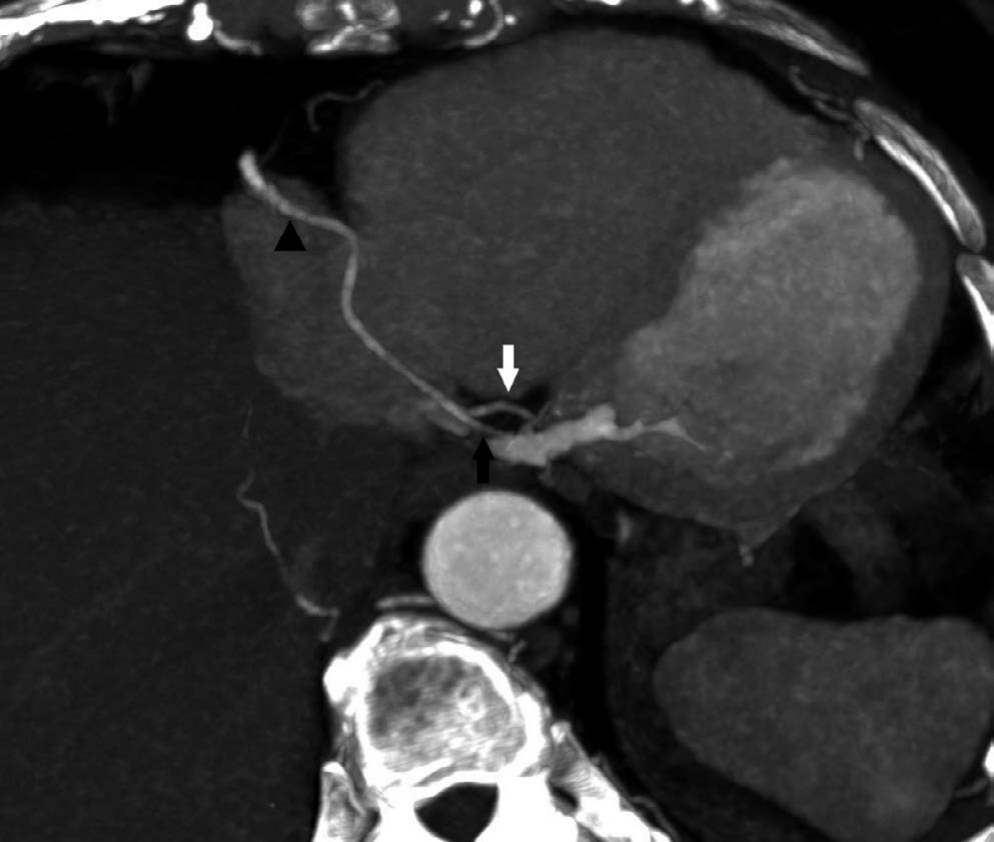
Axial image shows right coronary dominance. PDA (white arrow) & PLV (black arrow) arising from RCA (black triangle)

Computed tomography coronary angiography (CTCA) showed absent LCX in atrioventricular groove (Figure 1) with right coronary dominance and prominent RCA (right coronary artery) extending beyond right ventricular margin. No ectopic origin of LCX was identified. Normal origin of LCA (left coronary artery) was noted giving off prominent LAD (left anterior descending) artery and its first diagonal branches (Figure 2). The LCX territory was supplied by branches of PDA (posterior descending artery) & PLV (posterior left ventricular) and terminal branches of LAD (Figure 3). Total Calcium score was 0.4.

The patient was kept under observation for 24 hrs and discharged the following day under medical management and advised regular follow‐ups.

Congenital absence of LCX artery is a rare coronary artery anomaly with a reported incidence between 0.003% and 0.067%.^1^ Usually, it is benign and asymptomatic and discovered incidentally. It has been reported that congenital absence of the LCX might be associated with systolic click syndrome and could present with chest pain, episodic rapid heartbeats, and syncope^2^ which was observed in our patient. CTCA is currently the choice modality for detection of this anomaly over catheter angiography as it is less invasive and also helps in better delineating the course of the vessel in relation to cardiac chambers.^3^

While the absence of LCX has yet to be significantly associated with any major cardiac event, identification of this anomaly is crucial when performing cardiac interventions because such patients are at increased risk of being misdiagnosed during cardiac catheterization procedures, also they require extracare while performing cardiac bypass procedures to avoid accidental ligation or transection of anomalous vessels and to ensure that the grafts are placed properly to restore perfusion to ischemic myocardium.^4^, ^5^

## CONFLICT OF INTEREST

No conflict of interests to declare.

## **AUTHOR** **CONTRIBUTIONS**

BP: involved in initial drafting of manuscript. AS and AST: involved in review of the images. BK: involved in critical review of manuscript. All authors approved and finalized the manuscript.
